# Development and psychometric validation of the hospital Security Personnel Narrative Competency Scale (HSPNCS)

**DOI:** 10.1186/s12913-026-15542-7

**Published:** 2026-09-14

**Authors:** Binru Fang, Lina Guo, Hao Zhang, Yongting Yang, Rong Wu, Jin Wu, Shunjie Jiang, Liming Zhu

**Affiliations:** 1 Zhejiang Provincial Hospital Administration Center, Hangzhou, Zhejiang Province 310000 China; 2 Department of Quality Management, Zhejiang Cancer Hospital, Hangzhou, Zhejiang Province China; 3 School of Public Administration, Hangzhou Normal University, Hangzhou, Zhejiang Province China

**Keywords:** Narrative medicine, Medical humanities, Narrative competency, Hospital security personnel, Scale development, Healthcare support workforce

## Abstract

**Background:**

Hospital security personnel frequently interact with patients and families in emotionally complex and potentially conflict-prone situations. However, no specific instrument is available to assess narrative competency in this healthcare support workforce. This study aimed to develop and psychometrically validate the Hospital Security Personnel Narrative Competency Scale (HSPNCS).

**Methods:**

A methodological study was conducted to develop and validate the HSPNCS. Scale development followed a theory-driven adaptation approach based on the classical conceptualization of narrative competence and previously validated instruments. The initial item pool was generated through literature review, narrative medicine and medical humanities frameworks, the Nominal Group Technique, content validity assessment, and adaptation to the work context of hospital security personnel. A cross-sectional survey was then conducted among 645 hospital security personnel from 17 hospitals in Zhejiang Province, China. Psychometric evaluation included item analysis, content validity assessment, exploratory factor analysis, confirmatory factor analysis, convergent validity assessment, and internal consistency reliability testing.

**Results:**

The final HSPNCS consisted of 36 items organized into nine first-order subdomains nested within three higher-order dimensions: self-reflection, empathy, and professionalism. The scale demonstrated excellent internal consistency, with a Cronbach’s alpha of 0.967 for the total scale and alpha coefficients ranging from 0.911 to 0.945 for the three higher-order dimensions. Content validity was acceptable, with an S-CVI/Ave of 0.972. Factor analyses supported the proposed multidimensional structure, and confirmatory factor analysis indicated acceptable model fit. Composite reliability and average variance extracted values also supported the internal consistency and convergent validity of the scale.

**Conclusion:**

The HSPNCS demonstrated preliminary evidence of content validity, construct validity, convergent validity, and internal consistency among hospital security personnel. It may serve as a useful tool for assessing narrative competency and identifying training needs in this healthcare support workforce. Further studies using independent, multicenter, and culturally diverse samples are needed to confirm its factor structure, test–retest reliability, external validity, and responsiveness to training interventions.

**Supplementary Information:**

The online version contains supplementary material available at https://doi.org/10.1186/s12913-026-15542-7.

## Introduction

Narrative competency is a core component of narrative medicine, defined as the ability to identify, understand, and empathize with others’ disease and care narratives [1]. Narrative competency originates from clinical practice and aims to help healthcare professionals understand patients’ experiences, establish empathetic connections, and make more compassionate decisions [2, 3]. Over the past twenty years, interventions based on narrative competency have been shown to significantly improve communication between patients and healthcare providers, reduce medical disputes, and expand their value beyond clinical positions to various front-line personnel in the healthcare system [4, 5]. Therefore, narrative competency is a critical skill for all hospital staff who directly interact with patients and their families.

Hospital security personnel are a large but long-neglected group of front-line service providers in the global healthcare system. They face significant industry-wide challenges: in the United States, there are approximately 1.5 million hospital security personnel [6]. In China, hospitals often use third-party outsourcing for security services, but security personnel frequently interact with patients. Their role has expanded from traditional order maintenance to include patient guidance, handling emergencies, emotional support, and conflict resolution [7]. In their daily work, they frequently encounter anxious family members, emotionally disturbed patients, and various conflicts. Their narrative skills directly impact patient experience, the safety atmosphere in hospitals, and the stability of the doctor-patient relationship [8, 9]. However, this group is often marginalized. They lack training, have weak service awareness, and have limited sense of professional identity. For example, a study at a university hospital in Egypt found that workplace violence experienced by security personnel in healthcare settings is a serious issue, yet related training programs are lacking [10]. From 2015 to 2021, the Centers for Disease Control and Prevention (CDC) reported over 400,000 emergency department visits involving violence between security or law enforcement personnel and patients, which has led to frequent interpersonal conflicts and exacerbated the already tense relationship between patients and healthcare providers [11]. In China, the outsourcing model has further exacerbated the lack of service awareness and weak sense of professional identity among security personnel, which directly impacts the overall service quality of hospitals [12]. According to a national survey of patient complaints in Chinese hospitals, a significant proportion of complaints in the non-clinical staff category are related to poor attitude and ineffective communication [13, 14]. Therefore, enhancing the emotional intelligence, empathy, and conflict resolution skills of hospital security personnel has become a pressing issue and urgent need in the field of medical management.

The ability to empathize, actively listen, and comprehend others’ emotions, as well as respond effectively to their concerns, aligns closely with the core competencies required of a security officer in a medical facility. This includes effective communication in crisis situations and addressing the needs of service recipients. For security personnel in medical facilities, possessing strong communication skills allows them to more effectively address the needs of service recipients, de-escalate tensions, and enhance their sense of professional fulfillment [15]. However, the current tools used to assess these skills, such as the Jefferson Empathy Scale [16] and the Clinical Nurse Leader Empathy Scale [17], are designed for healthcare professionals and do not align with the needs of security personnel. Firstly, these tools depend on written scenarios and in-depth reflection, which do not reflect the spontaneous and crisis-oriented nature of security work [18]. Secondly, due to the varying educational levels among security personnel, these tools may not be suitable for them. Additionally, these tools do not address situations specific to security roles, such as conflict resolution and crowd management [19], and therefore lack the necessary specificity.

To address this research gap, this study aims to develop and validate the psychometric validity of the Hospital Security Personnel Narrative Competency Scale (HSPNCS). Based on the theory of narrative medicine, the scale integrates the job responsibilities of hospital security personnel with qualitative feedback from frontline security staff, hospital administrators, and patient representatives to construct a multidimensional assessment system. Through item generation, content validity testing, exploratory factor analysis (EFA), confirmatory factor analysis (CFA), and reliability testing, this study developed the HSPNCS, a 36-item scale comprising three higher-order dimensions and nine first-order subdomains. The HSPNCS may provide a quantitative tool for assessing narrative competency among hospital security personnel and for identifying training needs related to communication, empathy, self-reflection, and professional identity. It may also support future research on humanistic service improvement among non-clinical healthcare support staff.

## Methods

This study was designed as a methodological study involving the development and psychometric validation of the HSPNCS.The English translation of the HSPNCS is provided in Supplementary File 1 for reference. A cross-sectional survey was used during the psychometric testing phase.The research protocol received approval from the Medical Ethics Committee of Zhejiang Cancer Hospital, ensuring that all procedures were in full compliance with the principles outlined in the Declaration of Helsinki. Informed consent was obtained from all participants prior to their inclusion in the study, and participation was entirely voluntary. The study was ethically reviewed and approved under the reference number IRB-2023–749 (IIT).

**Fig. 1:**
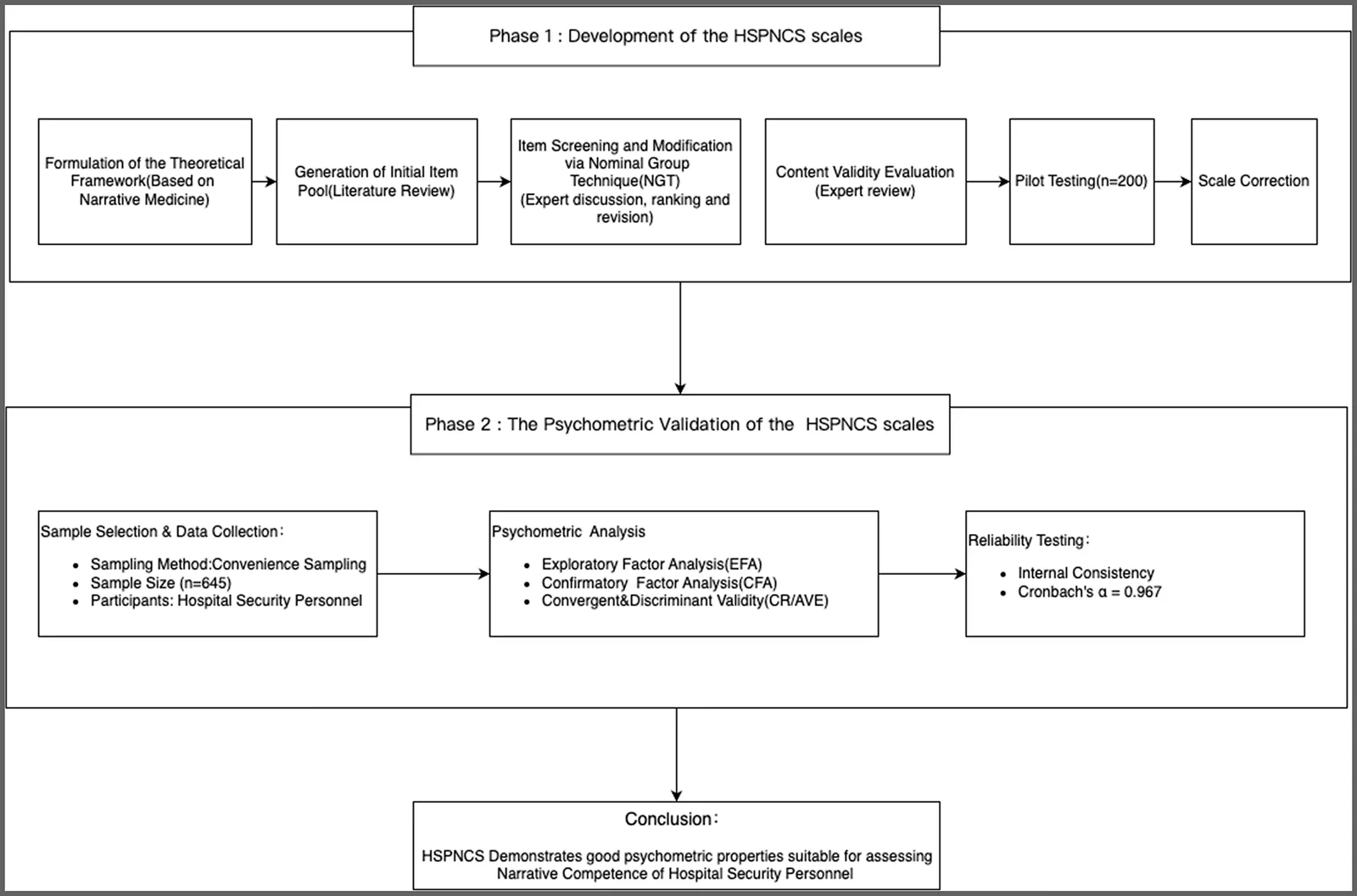
Research process for developing the hospital security personnel narrative competency scale (HSPNCS)

The overall workflow of scale development and psychometric validation is presented in Fig. 1. Briefly, the study comprised two sequential phases: Phase I involved conceptual framework development, item generation, expert review using the Nominal Group Technique, content validity assessment, pilot testing, and scale refinement; Phase II involved participant recruitment, data collection, and psychometric evaluation, including item analysis, exploratory factor analysis, confirmatory factor analysis, convergent validity assessment, and reliability testing.

### Phase I: development of the HSPNCS scales

#### Theory-driven conceptual framework and operationalization

This study reviews the international frameworks and measurement dimensions of narrative medicine. Most of these scales focus on assessing the competencies of physicians and nurses, and typically include dimensions such as empathy, reflection, trust, and cultural competence. However, directly applying these scales to hospital security personnel presents certain challenges for two reasons: first, the duties of hospital security personnel differ from those of healthcare professionals, as they rely more on verbal communication than written expression; second, there are significant variations in the professional backgrounds and educational levels of security personnel. Therefore, scale design must align with the practical needs of this diverse workforce. To enhance the scale’s applicability to hospital security personnel, the concepts to be measured must first be comprehensively defined to ensure that the scale’s items fully cover the content of these concepts and their relevant dimensions.

Drawing on the classical conceptualization of narrative medicine, particularly its emphasis on empathy, reflection, profession, trust, attention, representation, and affiliation, this study first identified self-reflection and empathy as two core domains of narrative competency. However, hospital security personnel differ from clinical professionals in their work responsibilities, educational background, and modes of interaction with patients and families. Their narrative competency is expressed not only through reflective awareness and empathic understanding, but also through their occupational identity, perceived work value, and service-oriented commitment in daily security practice. Therefore, professionalism was incorporated as a context-specific extension of the narrative medicine framework rather than as a direct component of the original classical definition. Accordingly, the preliminary framework of the HSPNCS comprised three higher-order dimensions: self-reflection, empathy, and professionalism.

Definitions of the three higher-order dimensions of the HSPNCS are provided in Table 1.

**Table 1:** Definitions of the three higher-order dimensions of the HSPNCS

Dimension	Description
Self-Reflection	It refers to the process of thinking repeatedly and introspectively. It involves thinking about the emotional, social, and personal elements encountered in healthcare and understanding how these context affect patients and healthcare providers.
Empathy	The capacity to listen to another person’s narrative and to adopt a perspective that is analogous to that of the other individual
Professionalism	It refers to the inner passion of an individual who has a strong sense of value for a particular occupation.

#### Development of the measurement item pool

##### Item extraction from literature and interviews

To ensure conceptual clarity and psychometric rigor, item generation followed a theory-guided adaptation strategy rather than developing all items de novo.The item pool was constructed based on a literature analysis using databases such as CNKI, Medline, PubMed, Web of Science, Springer Link. The pool of items includes topics such as Narrative Medicine, Narrative-based Medicine, Narrative Competency, Narrative Nursing, Hospital Security Personnel, Self-Reflection, Empathy, Professionalism, and so on.

In this study, we conducted a comprehensive review of the existing literature to identify representative items suitable for our research context, with the aim of providing a more comprehensive representation of the concept’s scope. Given the unique nature of the hospital security personnel population, this study aims to develop an assessment tool that is both broadly applicable and highly targeted, capable of accurately evaluating hospital security personnel’s emotional empathy and cognitive understanding in patient care and daily communication, while ensuring the tool is easy to implement and disseminate. Therefore, we prioritized validated assessment items with established applicability and reliability, and selected the most appropriate assessment content based on the specific connotations of each assessment dimension.

The validated source instruments, corresponding operational subdomains, and rationale for their adaptation to the HSPNCS are summarized in Table 2.

**Table 2:** Validated source instruments and operational subdomains of the HSPNCS

Higher-order dimension	Source instrument	Operational subdomains in HSPNCS	Rationale
Self-Reflection	the Self-reflection and Insight Scale (SRIS) (self-reflection dimension) (developed by Grant et al.) [20]	ES, NS, PT	Items are neutral and easy to use or adapt for use in different cultural contexts. Their good applicability and reliability have been demonstrated. It is easy to administer and suitable for a group of security personnel with relatively different educational backgrounds.
Empathy	Chinese version of the Interpersonal Reactivity Index-C (developed by Zhang Zhiyu et al.) [21]	FS, EC, PD	Compared to more specialized scales such as the JSE [16], the IRI-C has a more generalized set of items that can accurately capture the emotional empathy and cognitive understanding of hospital security personnel in patient care and daily communication. The IRI-C’s 22 items are relatively brief and easy to administer and disseminate, making it particularly suitable for security personnel with varying educational levels and cognitive abilities.
Professionalism	Professional Sense of Mission Scale (developed by Zhang Chunyu) [22]	AC, MV, GF	It reflects the characteristics of the professional sense of mission in the context of Chinese society more than the foreign-developed scales (such as the CVQ [23]), especially in terms of the emphasis on the Altruistic Contribution dimension. In particular, the emphasis on the “Altruistic Contribution” dimension helps to assess the sense of professional responsibility of security personnel in crisis management and patient care. This directly reflects the individual’s perception of professional significance and is highly consistent with the goal of constructing the Narrative competency Scale.

##### Nominal group technique (NGT)

To ensure the face validity and content validity of the occupational trait assessment scale for hospital security personnel, this study convened a 6-member expert panel and employed the Nominal Group Technique (NGT) to qualitatively evaluate and screen the initial item pool [24]. The panel ensured diverse representation and multidimensional perspectives, comprising two narrative medicine researchers, two hospital administrators, one security supervisor, and one patient representative.

The NGT strictly followed a four-step structured protocol: (1) Silent generation: each expert independently reviewed the initial 45 items; (2) Round-robin recording: experts sequentially expressed their opinions on the relevance of each item to the research constructs and the clarity of its phrasing; (3) Serial discussion: discrepancies were discussed in-depth, and ambiguous or confusing contents were merged or rephrased; (4) Anonymous voting: based on the consensus achieved in the previous three steps, experts voted to determine the final retention or exclusion of the items.

Based on the anonymous voting results and expert consensus, 3 initial items were eliminated in this phase. The primary justifications for exclusion were categorized into two aspects: First, Item 20 leaned towards leisure and entertainment scenarios, failing to closely align with the specific context of security work. Second, conceptual redundancy or the risk of social desirability bias. Item 12 overemphasized external evaluation.

Following the rigorous qualitative screening via NGT, the aforementioned 3 items were excluded, leaving 42 highly suitable items to proceed to the subsequent quantitative Content Validity Index (CVI) evaluation phase.

#### Language adaptation and refinement

In light of previous studies, certain questions were adapted to align with the doctor - patient environment and the job duties of the security personnel at the research venue. The initial questions underwent a purification process through the expert calibration and assessment method, resulting in the following main modifications:

First, the reverse-scored items were transformed into positively worded items. For example, “I don’t often think about my thoughts” became “I often think about my thoughts”. The rationale behind this change is twofold. Firstly, the understanding and reaction ability of hospital security personnel is more suitable for positive questioning. Secondly, the question items are substituted with the roles of hospital security personnel. For example, the item “I want to engage in a job that contributes to society” is changed to: “Working as a security guard contributes to society.” The third approach is to modify the question items that are unclear or susceptible to ambiguity by combining the Chinese expression and the content of security work in hospitals. For instance, the item “Before criticizing others, I try to imagine: if I were in his situation, how would I feel?” can be rewritten as “Sometimes I am unable to control my anger towards others, but at this time I will consider how I would feel if I were in their position”.

The wording of some questions was deemed too esoteric for hospital security staff, necessitating the optimization of the semantic context and the addition of specific examples. To illustrate, the sentence “After watching a play or movie, I feel as if I were one of the characters in the play” was modified to “After reading a novel, or watching a movie, or a short video (e.g. Douyin, Kuaishou, etc.), I feel as if I were one of the characters in it” to assist security personnel in comprehending the intended meaning. Finally, we also invited a hospital security staff to read and complete the questionnaire. By observing his reactions while reading, we confirmed that the scale aligns with the cognitive and comprehension abilities of hospital security guards.

#### Pilot testing

Prior to the formalization of the scale, a preliminary investigation was conducted. This entailed the administration of an online questionnaire to 200 hospital security personnel. The objective was to ascertain the reasonableness of the scale and to identify any potential issues. The questionnaire was subjected to reliability and validity testing, with the results analyzed to inform modifications to the scale.

#### Scale correction

A preliminary analysis of the reliability and validity of the premeasurement scale revealed that the standardized factor loading value of several items was below 0.7, indicating a weak correspondence between the item and the factor. Consequently, 6 items were deleted, and the remaining indicators were recoded.

### Phase II:The psychometric validation of the HSPNCS scale

#### Participant recruitment and data collection

In China, hospital security management generally follows national requirements for the construction of hospital safety and order systems. According to the national guideline, hospitals should allocate security guards according to the highest applicable standard among no fewer than 3% of on-duty medical staff, one security guard per 20 beds, or 3‰ of the average daily outpatient volume. Hospitals are also required to establish security monitoring centers, integrate video surveillance, intrusion alarms, access control, and electronic patrol systems, and ensure video surveillance coverage of public and key areas. In addition, one-button alarm devices should be installed in gatehouses, departments, and key locations and connected to the hospital security monitoring center. In the present study, participating hospitals were therefore expected to operate within this general national security framework.

Data were collected in 18 hospital districts of 17 hospitals, including specialty and general hospitals.The participating hospitals were selected to include institutions from different geographic regions of Zhejiang Province. To ensure a comprehensive representation of regional disparities in healthcare resource allocation, hospitals were sampled from cities across distinct geographical regions of Zhejiang Province, including the eastern region (Hangzhou, Ningbo), the southern region (Wenzhou), the western region (Lishui), and the northern region (Jiaxing, Shaoxing, Jinhua). Inclusion criteria were as follows: hospital security personnel who provide services directly to patients; were on duty on the day of the survey and had been working in the survey unit for more than six months; and were willing to participate in and agree to the study. Details of the surveyed hospitals are presented in Table 3.

**Table 3:** General information and HSPNCS scales of surveyed hospitals

Hospital	Area	Building Area(m^2^)	Final Sample (n)	Eligible Staff (N)
Zhejiang University School of Medicine Affiliated First Hospital – Zhijiang Campus	Hangzhou	180000	78	130
Boao Campus of the Second Affiliated Hospital of Zhejiang University School of Medicine	Hangzhou	73800	36	40
Affiliated Cancer Hospital of Hangzhou Medical Research Institute, Chinese Academy of Sciences (Zhejiang Cancer Hospital)&Hangzhou Institute of Medical Sciences, Chinese Academy of Sciences	Hangzhou	58983&80000	87	139
Xixi Campus of Zhejiang Provincial Hospital of Traditional Chinese Medicine	Hangzhou	64000	23	43
Xiasha Branch of Zhejiang Provincial Hospital of Traditional Chinese Medicine	Hangzhou	46667	55	94
second affiliated hospital of zhejiang university of chinese medicine (zhejiang xinhua hospital)	Hangzhou	83333	26	48
Hangzhou Second People’s Hospital	Hangzhou	35829	48	83
Hangzhou Ninth People’s Hospital	Hangzhou	84667	36	44
Hangzhou Red Cross Hospital	Hangzhou	38000	29	46
Hangzhou Maternity and Gynecology Hospital	Hangzhou		48	67
Wenzhou Central Hospital	Wenzhou	33000	9	34
Hangzhou Fuyang Traditional Chinese Medicine Orthopedic Hospital	Hangzhou	60000	32	46
Haining (Jiaxing)Central Hospital	Jiaxing	70000	20	37
Ningbo Pediatric Hospital	Ningbo	42000	16	37
Zhuji (Shaoxing)Traditional Chinese Medicine Hospital	Shaoxing	70000	39	80
Yiwu (Jinhua)Central Hospital	Jinhua	177000	47	88
Lishui Hospital of Traditional Chinese Medicine	Lishui	19467	16	30

Participants completed the questionnaire either through an online link accessed by QR code or by using a paper-and-pencil format.The purpose and significance of the study were introduced to the respondents using a standardized guideline, and the questionnaire was completed after signing the informed consent.Online responses were collected anonymously through the Wenjuanxing platform (wjx.cn).For offline respondents, timely on-site communication was conducted throughout the survey administration (18 participants in total). Some participants encountered visual impairment due to presbyopia and were unable to clearly read questionnaire items, while others had limited literacy in Chinese characters. For respondents facing questionnaire completion barriers, trained investigators only offered procedural guidance, including instructions on how to fill out the questionnaire; no explanatory interpretation of item connotations, answer hints, or proxy filling were provided.

Participants responded to a questionnaire that included a sociodemographic section (including age, gender, family situation, etc.) and the HSPNCS. The HSPNCS is a newly developed 36-item scale consisting of nine first-order subdomains nested within three higher-order dimensions: self-reflection, empathy, and professionalism.Participants were asked to rate a Likert 5-point scale from 1 (strongly disagree) to 5 (strongly agree) to describe how much they agreed with each statement. Basic information of the interviewee is presented in Table 4.

**Table 4:** Participants’ demographic and occupational characteristics

Category		Number of Hospital Security Personnel	Percentage (%)
Gender	Male	564	87.4
	Female	81	12.6
Age	18 ~ 29	119	18.4
	30 ~ 39	129	20.0
	40 ~ 49	174	27.0
	50 ~ 59	214	33.2
	60+	0	0.0
Years of experience	1 ~ 9	488	75.7
	10 ~ 19	102	15.8
	20 ~ 29	36	5.6
	30 ~ 39	14	2.2
	40+	5	0.8
Region	Eastern region	514	79.7
	South region	9	1.4
	Western region	16	2.5
	North region	106	16.4

#### Psychometric analysis

Descriptive statistics as means, standard deviations, and percentages were used to describe the demographic data of the sample. The psychometric evaluation followed a structured multi-step validation procedure:

Item Analysis: Prior to factor extraction, item analysis was conducted using the item-total correlation method. The correlation coefficient of each item with the total scale score was calculated, and items with a correlation coefficient of less than 0.40 were deleted.

Content Validity: Content validity was evaluated by a 6-member expert panel using the Content Validity Index (CVI) based on the Polit and Beck (2006) framework. Experts rated the relevance of each item on a 4-point Likert scale. An Item-Level CVI (I-CVI)≥0.78 and a Scale-Level Average CVI (S-CVI/Ave)≥0.90 were established as the robust thresholds for retaining items.

Construct Validity: Exploratory factor analysis and confirmatory factor analysis were used to assess construct validity.

For the EFA, data was preliminary evaluated using the Kaiser-Meyer-Olkin test (KMO > 0.80) [25], the Bartlett test of sphericity (*p* < 0.05), These results indicated that the data were suitable for factor analysis. Using principal component analysis as the extraction method, and applied the oblimin to select oblique rotation. The criteria for including each item were based on a primary factor loading of >|0.50| and a meaningful and useful contribution to the target factor.

For the CFA, the maximum likelihood method was used to estimate the model. The relevant fitting indicators were selected to judge the model fit, which were mainly examined from absolute fitting indicators (χ^2^/*df*, RMSEA) and incremental fitting indicators (NFI, CFI).The chi-square value (χ^2^) is an indicator to judge the overall model fit, which is used to assess the size of the difference between the covariance matrices of the samples, and the smaller the CMIN is, the better the theoretical model fits the actual data; the smaller the chi-square to the degrees of freedom ratio (χ^2^/*df*) is, the better the covariance matrices of the theoretical model fits the actual data and the better the fit, and it is usually required to have a χ^2^/*df*﹤3, RMSEA is the root mean square of the approximation error, and its value ranges from 0.05 to 0.08 means that the model has a good fit. AGFI is the adjusting the goodness-of-fit index, NFI is the normal fit index, and CFI is the comparative fit index, and the two indexes ﹥0.9 means that the model has a good fit [26]. Data are non-normally distributed. The z-statistic was 292.505, which indicates non-normality of the data. Thus, in order to accommodate the lack of multivariate normality, we applied the Bollen-Stine bootstrap method to adjust the model and parameters [27]. The model was accepted as the results of fitting parameters. It showed that the *p* value of each path was less than 0.05, then the model was modified with correction index.

Convergent validity and internal consistency of the latent constructs were evaluated based on Confirmatory Factor Analysis (CFA). Internal consistency was assessed via the composite reliability (CR) coefficient, where a standard threshold of CR ≥ 0.70 indicates adequate reliability. Convergent validity was established using two standardized criteria: (1) item standardized factor loadings exceeding the robust benchmark of 0.50, and (2) Average Variance Extracted (AVE) values surpassing the 0.50 threshold, which demonstrates that the latent construct accounts for more than 50% of the variance in its designated observable indicators.

Internal consistency reliability was assessed using Cronbach’s alpha, with values ≥ 0.70 considered acceptable. Composite reliability (CR) was also calculated, with CR ≥ 0.70 indicating adequate reliability.

The significance level was set at *p* < 0.05(two-tailed). Data analyses were conducted using IBM SPSS Statistics version 26.0 and IBM SPSS AMOS version 26.0.

## Results

### Item analysis

After the NGT and content validity assessment, the 42-item preliminary version was administered in the pilot test. 6 items with standardized factor loadings below 0.70 were removed, resulting in the final 36-item version for formal psychometric validation. In the formal validation sample, corrected item-total correlations for all 36 items exceeded the preset threshold of 0.40; therefore, no additional items were removed at this stage.

### Content validity

Based on the content validity evaluation framework proposed by Polit and Beck (2006), a quantitative validation was conducted on the 42 items retained after the NGT screening.6 experts were invited,including academics and hospital administrators, to form an evaluation panel.The expert panel independently assessed each item in terms of relevance, clarity, and representativeness in relation to the target construct.Data analysis showed that the Item-Level Content Validity Index (I-CVI) for all items ranged from 0.833 to 1.000, meeting the acceptable threshold of 0.78. Consequently, all items were retained.

Regarding overall scale validity (as shown in Table 5), the Universal Agreement Scale-Level CVI (S-CVI/UA) was 0.833, meeting the ≥0.80 criterion. The Average Scale-Level CVI (S-CVI/Ave) was 0.972, fulfilling the ≥0.90 standard. These results indicate that the preliminary HSPNCS demonstrated adequate content validity.

**Table 5:** Scale-level content validity index (S-CVI) of the assessment scale

Metric	Value	Criterion	Evaluation
Universal Agreement S-CVI (S-CVI/UA)	0.833	Value ≥ 0.80	Pass
Average S-CVI (S-CVI/Ave)	0.972	Value ≥ 0.90	Pass

### Descriptive statistics

A total of 645 participants completed the survey. The mean age was 45.0 years (SD = 11.39; range: 18–59). Most participants were male (*n* = 564, 87.4%). Participants were predominantly from the eastern region of Zhejiang Province (*n* = 514, 79.7%). The majority had 1–9 years of work experience (*n* = 488, 75.7%).

### Construct validity

#### Exploratory factor analysis

A dimensionality reduction analysis was conducted on all the entries with a KMO value of 0.962 and a Bartlett’s test of sphericity approximate chi-square of 19,062.76 (*p* < 0.001), indicating that there are more common factors among the entries, which renders them suitable for factor analysis, and the results are shown in Table 6.

These values indicate strong inter-item correlations and substantial shared variance, confirming that the data is highly suitable for factor analysis. Subsequently, principal component analysis (PCA) was employed for factor extraction, and oblimin rotation was applied because the subdomains were expected to be correlated. The exploratory factor analysis supported a nine-factor first-order structure. These nine factors corresponded to the predefined subdomains and were conceptually grouped under three higher-order dimensions: self-reflection, empathy, and professionalism.

**Table 6:** Rotated factor loadings of the HSPNCS items

Item NO.	Rotated factor loadings								
	ES	NS	PT	FS	EC	PD	AC	MV	GF
ES1	0.767								
ES2	0.823								
ES3	0.745								
NS1		0.663							
NS2		0.861							
NS3		0.69							
PT1			0.753						
PT2			0.699						
PT3			0.744						
PT4			0.761						
PT5			0.769						
FS1				0.739					
FS2				0.805					
FS3				0.804					
FS4				0.773					
FS5				0.673					
EC1					0.753				
EC2					0.746				
EC3					0.735				
EC4					0.637				
EC5					0.789				
PD1						0.762			
PD2						0.83			
PD3						0.877			
PD4						0.843			
PD5						0.788			
AC1							0.771		
AC2							0.807		
AC3							0.604		
AC4							0.703		
MV1								0.841	
MV2								0.869	
MV3								0.748	
GF1									0.833
GF2									0.581
GF3									0.676

Content-wise, these factors aligned with the previously established dimensions, elucidating the scale’s internal structure and demonstrating that the proposed model possesses a high degree of theoretical fit. The rotated factor matrix exhibited a distinct “simple structure.” All 36 items loaded precisely onto their designated factors, with no notable cross-loadings observed. Furthermore, the standardized factor loadings for all items on their primary factors ranged from 0.581 to 0.877, consistently exceeding the recommended robust threshold of 0.50.

#### Confirmatory factor analysis

The study tested the construct validity using CFA. The results in Table 7 indicated that the normed chi-square (${\chi ^2}/df = 1.785$) met the model fitness criteria, and the fitness indicators (GFI = 0.944, AGFI = 0.931, CFI = 0.975, RMSEA = 0.035) were within the acceptable range. Additionally, all standardized factor loadings were statistically significant, and the model fit indices indicated an acceptable fit. No substantial localized misfit was observed after theoretically justified model modification

**Table 7:** Fitting results of the structural equation model (*n* = 645)

Fitting index	Fitting standard	Model	
		Initial model	Modified model
Chi-square freedom ratio χ^2^/*df*	1 < χ^2^/*df* < 3 Good	3.013	1.785
Root mean square error of approximation *RMSEA (90%CI)*	<0.05 Good	0.056	0.035
Goodness of fit index, *GFI*	>0.90 Good	0.870	0.944
Adjusted goodness of fit index, *AGFI*	>0.90 Good	0.845	0.931
Normed fit index, *NFI*	>0.90 Good	0.906	0.944
Confirmatory fit index, *CFI*	>0.90 Good	0.935	0.975
Bentler and Bonett’s non-normed fit index, *TLI*	>0.90 Good	0.927	0.971

#### Convergent validity

As shown in Table 8, the study further examined the scale’s composite reliability and convergent validity. In general, a composite reliability (CR) ≥0.70 indicates adequate internal consistency. The table shows that the composite reliabilities of all nine sub-factors ranged from 0.812 to 0.909, indicating that the scale has strong internal consistency. Furthermore, the analyses indicate that all items have factor loadings ranging from 0.726 to 0.871 (all ≥ 0.50), and the AVE values exceed 0.50 (ranging from 0.590 to 0.730), suggesting robust convergent validity for the scale.

**Table 8:** Measurement items and results of reliability and validity analysis of the questionnaire (*N* = 645)

Construction	Latent Variable	Measurement items	Load^a^	CR	AVE
Self-Reflection	ES	ES1	0.779	0.846	0.648
		ES2	0.815		
		ES3	0.82		
	NS	NS1	0.743	0.812	0.590
		NS2	0.753		
		NS3	0.807		
Empathy	PT	PT1	0.817	0.899	0.641
		PT2	0.832		
		PT3	0.866		
		PT4	0.74		
		PT5	0.741		
	FS	FS1	0.778	0.892	0.623
		FS2	0.793		
		FS3	0.835		
		FS4	0.806		
		FS5	0.731		
	EC	EC1	0.839	0.909	0.667
		EC2	0.819		
		EC3	0.851		
		EC4	0.739		
		EC5	0.83		
	PD	PD1	0.726	0.900	0.644
		PD2	0.816		
		PD3	0.849		
		PD4	0.854		
		PD5	0.76		
Professionalism	AC	AC1	0.741	0.873	0.633
		AC2	0.807		
		AC3	0.776		
		AC4	0.853		
	MV	MV1	0.857	0.890	0.730
		MV2	0.871		
		MV3	0.835		
	GF	GF1	0.761	0.856	0.665
		GF2	0.856		
		GF3	0.826		

all-load values are significant at the 0.001 level; CR: composite reliability; AVE: average variance extraction

*p* < 0.001, two-tailed test

### Reliability

As shown in Table 9, the Cronbach’s α coefficient of the HSPNCS was 0.967, and the Cronbach’s α coefficients of the factors were 0.911, 0.932, and 0.945, respectively, which indicated that there was good internal consistency of the scale.

**Table 9:** HSPNCS Cronbach’s α

HSPNCS	Item	Factor	Cronbach’s α
HSPNCS Cronbach’s α = 0.967	Self-Reflection Cronbach’s α = 0.911	ES（Engagement in self-reflection)	0.864
		NS（Need for self-reflection)	0.812
		PT（Perspective Taking)	0.898
	Empathy Cronbach’s α = 0.932	FS（Fantasy)	0.891
		EC（Empathic Concern)	0.907
		PD（Personal Distress)	0.899
	Professionalism Cronbach’s α = 0.945	AC（Altruistic Contribution)	0.871
		MV（Meaning and Value)	0.814
		GF（Guiding Force)	0.855

## Discussion and limitation

### Discussion

This study developed and preliminarily validated the HSPNCS through a theory-driven scale adaptation process. Rather than generating dimensions solely from empirical data, we first identified three higher-order domains of narrative competence—self-reflection, empathy, and professionalism—based on the classical conceptualization of narrative medicine.

The self-reflection dimension of the HSPNCS was operationalized by integrating three complementary constructs derived from previously validated instruments: engagement in self-reflection (ES), need for self-reflection (NS), and perspective taking (PT). These subdomains were selected because together they capture the behavioral, motivational, and interpersonal-cognitive aspects of reflective practice, which are central to narrative competence.The empathy dimension was adapted from the Interpersonal Reactivity Index (IRI), incorporating fantasy (FS), empathic concern (EC), and personal distress (PD) as three established subdomains of empathic responsiveness. These constructs were retained because they represent complementary cognitive and affective components of empathy that are highly relevant in emotionally charged hospital interactions.The professionalism dimension was operationalized through three constructs adapted from established occupational calling and professional identity scales: altruistic contribution (AC), meaning and value (MV), and guiding force (GF). These subdomains were selected to reflect the prosocial orientation, vocational significance, and value-guided behavioral commitment embedded in professional practice.

These domains were then operationalized by selecting and adapting constructs from previously validated instruments with established psychometric performance. As a result, the final HSPNCS contains 36 items organized into nine first-order subdomains nested within three higher-order dimensions. The factor analytic results supported this adapted multidimensional structure, suggesting that the selected constructs are conceptually meaningful and psychometrically applicable to hospital security personnel.While previous scholarship has overwhelmingly concentrated on operationalizing narrative medicine within the clinician-patient dyad [28, 29], the present research successfully extends this humanistic framework to the healthcare support workforce.

#### Psychometric robustness and dimensional structure

The final version of the HSPNCS comprises 36 items organized into nine first-order subdomains nested within three higher-order dimensions: self-reflection, empathy, and professionalism, demonstrated excellent internal consistency, with an overall Cronbach’s alpha of 0.967. This reliability coefficient is highly comparable to, and even exceeds, established clinical empathy and reflection instruments, such as the Jefferson Scale of Empathy with a Cronbach’s α of 0.81 for the total scale [30]. Conceptually, the emergence of self-reflection, empathy, and professionalism as the three empirical factors aligns seamlessly with Rita Charon’s seminal theoretical triad of narrative medicine—which posits that humanistic clinical practice rests upon “attention, representation, and affiliation” [31] Specifically, the *Self-Reflection* dimension captures the internal processing of emotionally charged encounters. As Mann et al. demonstrated in their systematic review, reflective capacity is the primary cognitive mechanism that buffers frontline healthcare workers against compassion fatigue and emotional exhaustion. For security personnel facing volatile environments, this introspective ability is vital for maintaining psychological resilience [32].

#### Extending the boundaries of narrative medicine

This scale is noteworthy as the first known scale specifically designed to assess the narrative competency of hospital security personnel. Prior research has centered on clinical staff such as doctors and nurses, while the role of support staff in fostering a positive healthcare environment remains under-explored. By extending the concept of narrative competency to hospital security personnel, this study broadens the scope of narrative medicine.

Our findings challenge the traditional, compartmentalized view of hospital administration, which often treats security outsourced personnel as mere physical deterrents. Modern health systems research indicates that the institutional “safety climate” is co-created by all initial points of contact [33]. Distressed patients and anxious family members do not differentiate between clinical and non-clinical staff during moments of crisis; their initial verbal de-escalation by a security officer directly sets the emotional trajectory for subsequent clinical encounters. Therefore, recognizing narrative competency as an essential skill for all staff fosters an inclusive and interdisciplinary approach to healthcare delivery.

#### Managerial implications and humanistic intervention

The findings of this study carry several significant implications for hospital management and healthcare delivery. Security personnel play a pivotal role in maintaining safety and order; nevertheless, their interactions with visitors have the potential to significantly impact patient satisfaction. It is imperative to cultivate the narrative abilities of security personnel to enhance these interactions.

The adoption of the HSPNCS enables healthcare organizations to not only evaluate the current proficiency of their security personnel but also to track their progression over time. Empirical evidence from clinical communication trials confirms that structured humanistic training significantly improves patient-reported experience scores [34]. The HSPNCS provides hospital administrators with the missing quantitative baseline required to design, implement, and longitudinally audit analogous communication curricula tailored to the unique, crisis-oriented workflows of security staff. Ultimately, the scale offers a pragmatic approach for transforming routine security management into an active component of hospital-wide narrative care.

### Limitations and future research

Notwithstanding the study’s merits, it is imperative to acknowledge its limitations. First, although hospitals from multiple regions and hospital types were included, the sample was restricted to hospital security personnel from 17 hospitals within a single province in China using a non-probability sampling approach. This geographic limitation and sampling method may restrict the generalizability of the findings. Therefore, future studies should further validate the HSPNCS across diverse healthcare settings and broader geographic regions to assess its cross-cultural applicability and external validity.

Second, the study relied primarily on self-reported measures, which may introduce social desirability and reporting biases despite anonymous administration. In addition, the use of a single-source self-report design may increase the risk of common method bias. Although self-reports reflect participants’ perceptions of their competencies, they may not fully correspond to actual behaviors in practice. Future research should incorporate observational approaches, such as observations of interactions between hospital security personnel and patients, to provide more objective assessments. Furthermore, investigator assistance was provided to participants with reading or writing difficulties, which may have introduced interviewer-related bias despite standardized training procedures.

Third, the quantitative cross-sectional design precludes causal inferences and the assessment of temporal stability. Future longitudinal studies are warranted to evaluate the test–retest reliability and long-term stability of the scale.

Finally, future research should further examine the relationship between hospital security personnel’s narrative competency and patient-related outcomes, including patient satisfaction and emotional well-being. Demonstrating whether enhanced narrative competency contributes to measurable improvements in patient outcomes may provide stronger empirical support for integrating narrative competency training into hospital security personnel development programs.

## Conclusion

This study developed the HSPNCS using a theory-driven adaptation approach based on the classical conceptualization of narrative competence and previously validated instruments. The final 36-item scale comprises nine first-order subdomains nested within three higher-order dimensions: self-reflection, empathy, and professionalism. The findings provide preliminary evidence of the content validity, construct validity, convergent validity, and internal consistency of the HSPNCS among hospital security personnel in Zhejiang Province, China. The scale may serve as a useful tool for assessing narrative competency and identifying training needs in this healthcare support workforce. Further studies using independent, multicenter, and culturally diverse samples are needed to confirm its factor structure, test–retest reliability, external validity, and responsiveness to training interventions [35].

## Electronic supplementary material

Below is the link to the electronic supplementary material.

Supplementary material 1

Supplementary material 2

## Data Availability

Generated Statement: The original contributions presented in the study are included in the article/supplementary material, further inquiries can be directed to the corresponding author/s.
