# Supplementary material for: Development and psychometric validation of the hospital Security Personnel Narrative Competency Scale (HSPNCS)
Source: BMC Health Serv Res. 2026 Sep 14;26:1275. doi: 10.1186/s12913-026-15542-7 (PMC13579745; doi:10.1186/s12913-026-15542-7)
Supplement: Supplementary file 1 [file 12913_2026_15542_MOESM1_ESM.docx]

Literature_Review

Firstly, during the literature review phase, existing international frameworks and evaluation dimensions for narrative medicine practices were collected, organised, and reviewed. These are outlined in Table 1.

Table 1. Narrative Medicine Practice Frameworks

| Framework | Dimensions or Content |
| --- | --- |
| Integrating narrative into clinical practice.[1] | Empathy, Reflection, Professionalism, Trustworthiness, Textual Skills |
| Developing reflective practices in healthcare.[2] | Participant Satisfaction, Relationship-Building, Empathy, Perspective-Taking and Reflection, Resilience and Burnout Detection/Mitigation, Confidence/Personal Accomplishment, Narrative Competence, Ethical Inquiry, Pedagogical Skills, Clinical Skills, Relevance to Work, Institutional Impact, and Cultural Competence. |
| Behavioral Indicator Framework for Narrative Competence[3] | Narrative Vision, Narrative Construction (including Narrative Listening, Narrative Understanding, Narrative Thinking, and Narrative Representation), Healthcare Relationships (including Empathy, Communication, Affiliation, and Intersubjectivity), and Narrative Healthcare Nursing (including Responsive Care, Balancing Behavior, and Healthcare Reflection). |
| Narrative Nursing Knowledge-Attitude-Behavior Questionnaire[4] | Knowledge, Attitude, Behavior |
| Narrative Competence Scale[5] | Attention and listening、understanding、reflection and reproduce |
| Assessing Narrative Medicine Competencies in Healthcare Students[6] | Reflective Thinking Scale for Healthcare Students and Providers(RTS-HSP)、Patient–Healthcare Provider Communication Scale(P-HCS)、Empathy Scale in Patient Care(ES-PC)、Analytic Narrative Medicine Writing Scoring Rubric (ANMWSR) |
| Narrative Literacy for Chinese Medical Practitioners[7] | Narrative attitudes, narrative knowledge, and narrative experience,  Narrative Judgment and Medical Decision-Making Behavior |
| narrative medicine ability[8] | Narrative Medicine Conceptual Awareness Competencies, Narrative Skills Techniques Competencies, Narrative Medicine Practice Competencies |

A review of the extant literature revealed that the development of previous scales has typically focused on the assessment of the professional competencies of physicians and nurses. These assessments typically encompass dimensions such as empathy, reflective capacity, trust, and cultural competence [9–13]. Nevertheless, the direct application of these scales to hospital security personnel remains problematic.

Following the clarification of the dimensions of the scale, a literature review and analysis of scales used in international practice that are related to these dimensions was conducted.

The Empathy Scale is a psychometric instrument designed to assess an individual's capacity for empathic response. Empathy can be defined as the capacity to listen to another person's narrative and to adopt a perspective that is analogous to that of the other individual[14]. This entails the security officer's capacity to empathize, comprehend, and resonate with the experiences disclosed by the patient, engaging in profound reflection on the narratives and experiences conveyed by the patient. The tenets of narrative medicine underscore the significance of the healthcare provider's active presence and attentive listening, as well as their capacity to receive and comprehend the patient's narrative. Both encompass cognitive empathy, which assesses an individual's ability to cognitively understand the emotions, thinking, and perspectives of others (such as perspective taking), and affective empathy, which measures an individual's emotional resonance and care for the emotions of others (such as empathic concern). Given the distinctive characteristics of the hospital security personnel group, it is essential that the empathy assessment instrument be applicable to this specific population and that the content of the entries can be understood by the general population. The Interpersonal Reactivity Index (IRI)[15], developed by Davis, has been a widely utilized instrument for measuring general empathy in adults. In light of these considerations, Zhan Zhiyu and Wu Jingji from Taiwan, China, undertook a revision of the IRI, resulting in the development of a 22-item scale (IRI-C) for the measurement of general empathy in adults' daily lives[16]. This revised scale is divided into four dimensions: viewpoint selection, imagination, Sympathetic concern and personal suffering, with good reliability and validity, were included in the revised scale, which also took into account Chinese cultural norms regarding emotional expression and social interactions. This was done to ensure that the scale would be more closely aligned with the emotional experience and social interactions typical of the Chinese context. The revised scale has been validated as an appropriate tool for assessing empathy in the Chinese population.The IRI-C was designed to assess empathy in general life and is applicable to a wide range of people, including laypersons. Compared to more specialized scales such as the JSE[17], the IRI-C has a more generalized set of items that accurately capture the emotional empathy and cognitive understanding of hospital security personnel in patient care and daily communication. In addition, the IRI-C's 22 items are relatively brief and easy to administer and disseminate, making it particularly suitable for security personnel of varying educational levels and cognitive abilities. The empathy scale frameworks and measurement dimensions are summarised in Table 2.

Table 2. The Empathy Scale Frameworks

| **Framework** | **Dimensions or Contents** |
| --- | --- |
| Jefferson Scale of Empathy (JSE)[17] | Perspective Taking, Compassionate Care, Empathic Concern |
| Toronto Empathy Questionnaire (TEQ)[18] | Emotional Empathy (including Emotional Reactivity, Perceived Emotions of Others) |
| Balanced Emotional Empathy Scale (BEES)[19] | Positive and Negative Emotional Responses |
| Interpersonal Reactivity Index (IRI)[15] | Perspective Taking, Fantasy, Empathic Concern, Personal Distress |
| Cognitive and Affective Empathy Scale (CAE)[20] | Cognitive Empathy, Affective Empathy |
| Integrating Narrative into Clinical Practice[1] | Empathy, Reflection, Professionalism, Trustworthiness, Textual Skills |
| Reflective Practices in Healthcare[21] | Empathy, Reflection, Perspective-Taking, Narrative Competence, Resilience, Ethical Inquiry, Cultural Competence, Pedagogical and Clinical Skills, Institutional Impact |

The Reflection Scale is a tool that measures the ability to reflect, which refers to the process of thinking repeatedly and introspectively[22].Reflection is critical in narrative medicine and involves thinking about the emotional, social, and personal elements encountered in healthcare and understanding how these contexts affect patients and healthcare providers. This study used a reflection scale to assess whether safety officers had developed the ability to think deeply about patients' stories and experiences. Internationally accepted reflection scales typically cover several dimensions, the most important of which are: reflective behavior, which reflects an individual's ability to reflect on his or her emotions, behaviors, and thoughts (such as SRIS's Self-Reflection)[23]; insight and cognition, which creates a clear perception of the outcome of the reflection process (such as Insight); and critical reflection, which is the ability to assess deeper issues and structural influences (such as RTS's Critical Reflection). In the United States, Marilyn's team conducted an experimental study of reflective practice education for nurses using the Self-reflection and Insight Scale (SRIS) developed by Grant et al. and achieved good reliability and validity. The SRIS was developed in 2002 to measure self-reflection and insight in adults in general, and its items are neutral and easy to use or adapt for use in different cultural contexts. In China, the SRIS has been used in a variety of fields and in different countries and regions, and its good applicability and reliability have been demonstrated. It is easy to administer and suitable for a group of security personnel with relatively different educational backgrounds. For this study, indicators of the self-reflection dimension of the scale were selected, including engagement in reflection (referring to the exploration of one's own thoughts, feelings, and behaviors) and need for reflection (referring to the analysis of one's own thoughts, feelings, and behaviors). The Reflection Scale frameworks and measurement dimensions are summarised in Table 3.

Table 3. The Reflection Scale Frameworks

| **Framework** | **Dimensions or Contents** |
| --- | --- |
| Self-Reflection and Insight Scale (SRIS)[23] | Self-Reflection (tendency to engage in reflection), Insight (awareness and clarity about one's thoughts, emotions, and behaviors) |
| Reflective Thinking Questionnaire(RTQ)[24] | habitual action, understanding, reflection, and critical  reflection |
| Reflective Functioning Questionnaire (RFQ)[25] | Certainty about Mental States  Uncertainty about Mental States |
| Reflective Practice Questionnaire(RPQ)[26] | reflection-in-learning |

Occupational Sense of Purpose Scale: Occupational Sense of Purpose refers to the inner passion of an individual who has a strong sense of value for a particular occupation[27]. Currently, the core dimensions of the international sense of purpose scale include personal meaning, i.e., most scales include an assessment of the meaning and purpose of the occupation (such as Calling of the CS[28]), Social Contribution, which emphasizes the positive impact of work on others and society (such as Prosocial Orientation of CVQ[29]), and Value Orientation, which measures an individual's perception of the intrinsic value of a career (such as Intrinsic Motivation of MAWS[30]) . Some of the scales have been revised locally, such as Zhang Chunyu's Professional Sense of Mission Scale[31], which covers the three dimensions of Orientation, Altruistic Contribution, and Meaning and Value, and was developed for the Chinese cultural context, emphasizing the social and collective orientation of occupational values, which reflects the characteristics of professional sense of mission in the context of Chinese society more than the foreign-developed scales (such as the CVQ[29]), especially the emphasis on the Altruistic Contribution dimension. In particular, the emphasis on the "Altruistic Contribution" dimension helps to assess the sense of professional responsibility of security personnel in crisis management and patient care, which directly reflects the individual's perception of professional significance, and is highly consistent with the goal of constructing the Narrative Competence Scale. The Occupational Sense of Purpose Scale frameworks and measurement dimensions are summarised in Table 4.

Table 4. The Occupational Sense of Purpose Scale Frameworks

| **Framework** | **Dimensions or Contents** |
| --- | --- |
| Calling Scale，CS[28] | Calling |
| Calling and Vocation Questionnaire (CVQ)[29] | Transcendent Calling  Prosocial Orientation  Value-Driven Behavior |
| Motivation at Work Scale，MAWS[30] | External Regulation  Identified Regulation  Introjected Regulation  Intrinsic Motivation |
| Professional Sense of Mission Scale (PSMS)[31] | Orientation, Altruistic Contribution, Meaning and Value |

**References**

1.Frank AW. Narrative medicine: Honoring the stories of illness. Lit Med. 2007;26(2):408-12.

2.Remein CD, Childs E, Pasco JC, Trinquart L, Flynn DB, Wingerter SL, et al. Content and outcomes of narrative medicine programmes: A systematic review of the literature through 2019. BMJ Open. 2020;10(1): e031568.

3.Chu SY, Wen CC, Lin CW. A qualitative study of clinical narrative competence of medical personnel. BMC Med Educ. 2020;20: 1-3.

4.Huang H, Liu YL. Research progress on the clinical application of narrative nursing. Zhonghua Nurs. 2016;51(2):196-200.

5.Ma WZ, Gu P, Zhang JJ, Yi HX. Development of the Narrative Competence Scale and its test of reliability and validity. Chin J Nurs. 2020;55(4):578-83.

6.Liao HC, Wang YH. Storytelling in medical education: Narrative medicine as a resource for interdisciplinary collaboration. Int J Environ Res Public Health. 2020;17(4):1135.

7.Yang XL, Jia YZ, Zhao CY, et al. Development and reliability/validity testing of the Narrative Competencies Scale for Medical Practitioners. Med Philos. 2023;44(21):39-44.

8.Nuerbiya W, Liu J. On the cultivation of medical narrative medicine ability in China. Med Philos. 2019;40(06):54-59.

9.Kangaslampi S, Garoff F, Peltonen K. Narrative exposure therapy for immigrant children traumatized by war: Study protocol for a randomized controlled trial of effectiveness and mechanisms of change. BMC Psychiatry. 2015;15(1):127.

10.Ma W, Gu P, Zhang J, Yi H. Development of the Narrative Competence Scale and its test of reliability and validity. Chin J Nurs. 2020;55(4):578-83.

11.Yang X, Jia Y, Zhao C, Jin H, Lin Z. Development and reliability/validity testing of the narrative competencies scale for medical practitioners. Med Philos. 2023;44(21):39-44.

12.Brockington G, Gomes Moreira AP, Buso MS, et al. Storytelling increases oxytocin and positive emotions and decreases cortisol and pain in hospitalized children. Proc Natl Acad Sci USA. 2021;118(22): e2018409118.

13.Zhang Y, Pi B, Xu X, Li Y, Chen X, Yang N. Influence of narrative medicine-based health education combined with an online patient mutual assistance group on the health of patients with inflammatory bowel disease and arthritis. Patient Relat Outcome Meas. 2020;13: 1-10.

14.Charon R. Narrative medicine: A model for empathy, reflection, profession, and trust. JAMA. 2001;286(15):1897.

15.Davis MH. A multidimensional approach to individual differences in empathy. JSAS Cat Sel Doc Psychol. 1980

16.Zhang FF, Dong Y, Wang K, et al. Reliability and validity of the Chinese version of the Interpersonal Reactivity Index-C. Chin J Clin Psychol. 2010;18(2):155-157.

17.Hojat M, Mangione S, Nasca TJ, et al. The Jefferson Scale of Physician Empathy：development and preliminary psychometric data. Educ Psychol Meas,2001;61(2):349-365.

18.Spreng RN, McKinnon MC, Mar RA, Levine B. The Toronto Empathy Questionnaire: scale development and initial validation of a factor-analytic solution to multiple empathy measures. J Pers Assess. 2009;91(1):62-71.

19.Mehrabian, A. (1996). Manual for the Balanced Emotional Empathy Scale (BEES).

20.Reniers RL, Corcoran R, Drake R, Shryane NM, Völlm BA. The QCAE: a Questionnaire of Cognitive and Affective Empathy. J Pers Assess. 2011;93(1):84-95.

21.Remein CDiF, Childs E,Pasco JC, et al. Content and outcomes of narrative medicine

programmes: a systematic review of the literature through 2019. BMJ Open2020;10: e031568.

22. Charon R. Narrative medicine: A model for empathy, reflection, profession, and trust. JAMA. 2001;286(15):1897.
23. Grant AM, Franklin J, Langford P. The self-reflection and insight scale: A new measure of private self-consciousness. Social Behavior and Personality: an international journal. 2002;30(8):821-35.
24. Kember D, Leung DYP, Jones A, Loke AY, McKay J, Sinclair K, (2000). Development of a Questionnaire to Measure the Level of Reflective Thinking. Assessment & Evaluation in Higher Education, 2000:25(4), 381-395.
25. Fonagy P, Luyten P, Moulton-Perkins A, Lee YW, Warren F, Howard S, Ghinai R, Fearon P, Lowyck B. Development and Validation of a Self-Report Measure of Mentalizing: The Reflective Functioning Questionnaire. PLoS One. 2016;11(7): e0158678.
26. Sobral DT. An appraisal of medical students' reflection-in-learning. Medical education.2000;34:182-187.
27. Zhang AY, Song LJ, Tsui AS, Fu PP. Employee responses to employment‐relationship practices: The role of psychological empowerment and traditionality. Journal of Organizational Behavior. 2014 Aug;35(6):809-30.
28. Dobrow SR, Tosti-Kharas J. Listen to Your Heart? Calling and Receptivity to Career Advice. Career Assessment, 2012:20(3), 264-280.

29.Dik BJ, Eldridge BM, Steger MF,Duffy RD. Development and Validation of the Calling and Vocation Questionnaire (CVQ) and Brief Calling Scale (BCS). Career Assessment.2012:20(3), 242-263.

30.Gagné M, Forest J, Gilbert MH, Aubé C, Morin E, Malorni A. The Motivation at Work Scale: Validation Evidence in Two Languages. Educational and Psychological Measurement, (2010):70(4), 628-646.

31.Zhang C.A study of career as a calling in China:Structure,Assessment,and its Relation to well-being. Ph.D. Xinan University; 2015.
