# Supplementary material for: Development and psychometric validation of the hospital Security Personnel Narrative Competency Scale (HSPNCS)
Source: BMC Health Serv Res. 2026 Sep 14;26:1275. doi: 10.1186/s12913-026-15542-7 (PMC13579745; doi:10.1186/s12913-026-15542-7)
Supplement: Supplementary file 2 [file 12913_2026_15542_MOESM2_ESM.docx]

**Supplementary File 1
Chinese-English Bilingual Version of the Hospital Security Personnel Narrative Competency Scale (HSPNCS)**

Questionnaire ID / 编号: [	]

| **中文说明** | **English instructions** |
| --- | --- |
| 各位保安兄弟姐妹：人生来就有感情，面对工作、面对生活，有压力也有幸福，与他人接触交流，有开心也可能有冲突，这些时候，我们都有自己的想法。为了解大家的想法和行为，我们开展安保人员叙事能力的问卷调查，欢迎您参加。预计需请您填写2-3次，每次填写将花费您6-10分钟左右的时间。本次调查问卷中可能会存在询问个人及家庭隐私的内容，系从事该研究收集数据而开展，所有资料严格保密，请不要有任何顾虑。答案也没有对错之分，您只需要如实作答即可。请您认真填写问卷，感谢您的支持！ | Dear Security Staff: Everyone has feelings. In work and in life, we all have pressure and happiness. When dealing with other people, there may be pleasant moments, but sometimes there may also be conflicts. At these times, everyone has their own thoughts and feelings. To better understand your thoughts, feelings, and behaviors at work, we are carrying out this survey on narrative competency among hospital security personnel. You may be asked to complete this questionnaire 2-3 times. Each time will take about 6-10 minutes. Some questions may involve personal or family information. These questions are only for research purposes. All your information will be kept strictly confidential. There are no right or wrong answers. Please answer honestly according to your real thoughts and experiences. Thank you for your support! |
| 安保人员叙事能力课题组 | Hospital Security Personnel Narrative Competency Research Group |

**Response options / 作答选项: 1 = 完全不符合 / Strongly disagree; 2 = 不符合 / Disagree; 3 = 不确定 / Not sure; 4 = 符合 / Agree; 5 = 完全符合 / Strongly agree**

| **No.** | **Higher-order dimension** | **First-order subdomain** | **中文条目** | **English translation** |
| --- | --- | --- | --- | --- |
| 1 | Self-reflection | Engagement in self-reflection (ES) | 我经常会去琢磨自己的想法 | I often think about my own thoughts. |
| 2 | Self-reflection | Engagement in self-reflection (ES) | 我经常反思自己的想法 | I often reflect on my own ideas. |
| 3 | Self-reflection | Engagement in self-reflection (ES) | 我经常回想我的感受 | I often look back on my feelings. |
| 4 | Self-reflection | Need for self-reflection (NS) | 我觉得去思考自己的想法蛮有意思的 | I think it is interesting to think about my own thoughts. |
| 5 | Self-reflection | Need for self-reflection (NS) | 对我来说，知道自己的想法是怎么来的很重要 | It is important for me to know where my thoughts come from. |
| 6 | Self-reflection | Need for self-reflection (NS) | 我很重视去了解自己的感受 | I care a lot about understanding my own feelings. |
| 7 | Self-reflection | Perspective taking (PT) | 在做决定前，我会站在不同角度考虑问题 | Before making a decision, I try to think about the problem from different angles. |
| 8 | Self-reflection | Perspective taking (PT) | 我会站在不同的观点和立场来看问题 | I try to look at things from different viewpoints and positions. |
| 9 | Self-reflection | Perspective taking (PT) | 有时候我会从别人的角度来看事情，以便更了解他们 | Sometimes I look at things from other people’s point of view so I can understand them better. |
| 10 | Self-reflection | Perspective taking (PT) | 当别人惹我生气时，我通常会试着从他/她的角度去想 | When someone makes me angry, I usually try to think from his or her point of view. |
| 11 | Self-reflection | Perspective taking (PT) | 有时忍不住要冲别人发火，但这个时候我会想：如果我是他，我的感受会怎么样 | Sometimes I cannot help getting angry at others, but at that moment I will think: “If I were him or her, how would I feel?” |
| 12 | Empathy | Fantasy (FS) | 看小说或电影或短视频（如抖音快手等）时，我会投入到小说或电影或短视频中人物的感情世界里 | When reading novels or watching movies or short videos, I often feel involved in the emotions of the people in the story. |
| 13 | Empathy | Fantasy (FS) | 看小说或电影或短视频（如抖音快手等）时，我通常会全身心投入 | When reading novels or watching movies or short videos, I usually become fully involved in the story. |
| 14 | Empathy | Fantasy (FS) | 看完小说或电影或短视频（如抖音快手等）之后，我会觉得自己好像是里面的某一个角色 | After reading or watching, I sometimes feel like I was one of the people in the story. |
| 15 | Empathy | Fantasy (FS) | 当看小说、看电影或短视频（如抖音快手等）时，我很容易站在某个角色的立场去感受他/她的心情 | When reading novels or watching movies or short videos, it is easy for me to feel things from a character’s point of view. |
| 16 | Empathy | Fantasy (FS) | 当我看小说、看电影或短视频（如抖音快手等）时，我会想到：如果这个故事发生在我身上，我会怎么样 | When reading novels or watching movies or short videos, I often think: “What would I do if this happened to me?” |
| 17 | Empathy | Empathic concern (EC) | 看到那些可怜的人，我经常有心软和想去帮助他们的感觉 | When I see people who are suffering, I often feel soft-hearted and want to help them. |
| 18 | Empathy | Empathic concern (EC) | 当看到别人碰到困难或问题时，我会为他们感到难过 | When I see others facing difficulties or problems, I feel sad for them. |
| 19 | Empathy | Empathic concern (EC) | 当看到别人受伤害时，我会想到要保护他们 | When I see someone being hurt, I feel I should protect them. |
| 20 | Empathy | Empathic concern (EC) | 我会在意别人的处境 | I care about other people’s situations. |
| 21 | Empathy | Empathic concern (EC) | 当看到别人遇到不公平的对待时，我会同情他们 | When I see others being treated unfairly, I feel sympathy for them. |
| 22 | Empathy | Personal distress (PD) | 在紧急状况中，我会感到担忧、害怕而难以平静 | In an emergency, I feel worried or afraid and find it hard to stay calm. |
| 23 | Empathy | Personal distress (PD) | 当我情绪非常激动的时候，我往往不知道怎么办才好 | When I am very emotional, I often do not know what to do. |
| 24 | Empathy | Personal distress (PD) | 处在紧张情绪的状况中，我会惊慌害怕 | In tense situations, I feel nervous and scared. |
| 25 | Empathy | Personal distress (PD) | 在发生紧急情况时，我会紧张得几乎无法控制自己 | When emergencies happen, I may become so nervous that I can hardly control myself. |
| 26 | Empathy | Personal distress (PD) | 当看到别人发生意外而急需帮助时，我会非常紧张 | When I see someone having an accident and needing help urgently, I feel very nervous. |
| 27 | Professionalism | Altruistic contribution (AC) | 我正在干一个能帮助别人的工作 | I am doing a job that helps other people. |
| 28 | Professionalism | Altruistic contribution (AC) | 我当保安对社会有贡献 | My work as a security guard contributes to society. |
| 29 | Professionalism | Altruistic contribution (AC) | 我很在意当保安能不能对社会和别人有帮助 | I care a lot about whether my work as a security guard can help society and other people. |
| 30 | Professionalism | Altruistic contribution (AC) | 我要通过保安这份工作去做些对社会有帮助的事 | Through my work as a security guard, I want to do something helpful for society. |
| 31 | Professionalism | Guiding force (GF) | 我感觉有一种无形的力量推动着我从事保安这份工作 | I feel there is an invisible force pushing me to do this security job. |
| 32 | Professionalism | Guiding force (GF) | 我受到某种力量的感召而选择了保安这份工作 | I feel called by some kind of force to choose this security job. |
| 33 | Professionalism | Guiding force (GF) | 我感觉自己一直会去追寻自己想要的工作 | I feel that I will keep searching for the kind of work I truly want. |
| 34 | Professionalism | Guiding force (GF) | 我觉得自己应该去做保安这个工作 | I feel that being a security guard is a job I should do. |
| 35 | Professionalism | Meaning and value (MV) | 我要在保安这个工作中寻找到自己存在的价值 | I want to find my own value through my work as a security guard. |
| 36 | Professionalism | Meaning and value (MV) | 我要找到一份能让我感到自己存在价值的工作 | I want to find a job that makes me feel my life has value. |

**Basic information / 基本信息**

| **No.** | **中文项目** | **English translation** |
| --- | --- | --- |
| 37 | 您的性别：A 男 B 女 | Gender: A. Male; B. Female |
| 38 | 您的年龄：____ 岁 | Age: ____ years |
| 39 | 您的工作年限：____ 年 | Years of work experience: ____ years |
| 40 | 您的最高学历：A 初中及以下 B 高中或中专 C 大专 D 大学本科及以上 | Highest education level: A. Junior high school or below; B. High school / vocational school; C. College diploma; D. Bachelor’s degree or above |
| 41 | 您的婚姻状况：A 未婚 B 已婚 C 离异 D 其他（如丧偶） | Marital status: A. Single; B. Married; C. Divorced; D. Other (e.g., widowed) |
| 42 | 您的子女状况：A 无子女 B 一个子女 C 两个及以上子女 | Children: A. No children; B. One child; C. Two or more children |
| 43 | 您是否有亲兄弟亲姐妹：A 有 B 无 | Do you have brothers or sisters? A. Yes; B. No |
| 44 | 您现在的居住情况：A 集体宿舍住宿 B 与家人一起居住 C 独居 | Current living situation: A. Living in a shared staff dormitory; B. Living with family; C. Living alone |
| 45 | 您从事保安工作之前是否有其他工作经历：A 有 B 无 | Before working as a security guard, did you have other work experience? A. Yes; B. No |
| 46 | 您除了在本单位从事保安工作外，是否还在其他单位兼职：A 是 B 否 | Besides your security work in this hospital, do you also have a part-time job in another workplace? A. Yes; B. No |

*Note: This bilingual supplementary file provides the original Chinese items and their English translations for reference. The Chinese version was used in the formal survey.*
